# PD38 RedETSA: History And Achievements Since Its Creation

**DOI:** 10.1017/S0266462325101712

**Published:** 2025-12-29

**Authors:** Vania Cristina Canuto Santos, Miriam Malen Hollmann, Flavia Ribeiro Salomon, Caroline Bregman, Alexandre Lemgruber

## Abstract

**Introduction:**

The Health Technology Assessment (HTA) Network of the Americas (RedETSA) is composed of health ministries, HTA agencies, regulatory authorities, and collaborating centers that exchange valuable cooperation and information to promote the HTA processes in the Americas region. Since its creation, the network has grown significantly, and several milestones have been reached.

**Methods:**

A retrospective review of HTA milestones in the region and RedETSA network achievements was conducted, covering its inception at the HTAi annual meeting in Rio de Janeiro, Brazil, in 2011 to the present. The review included: the number of member countries and institutions; milestones in HTA development, implementation, and institutionalization; key Pan American Health Organization (PAHO) resolutions supporting HTA; advances in the Regional Database of Health Technology Assessment Reports of the Americas (BRISA); the number of annual meetings; regional and international cooperation; and capacity building support in the region.

**Results:**

Following the approval of the CSP28.R9 resolution on HTAs and Health Decision-Making by the PAHO in 2012 and the WHA67.23 resolution (World Health Organization) in 2014, HTA was implemented or institutionalized in several countries. Since its inception, RedETSA has grown to include 21 member countries in the Americas, represented by 40 institutions. HTA capacity building was supported by 482 scholarships and courses since 2015, as well as 43 webinars by RedETSA. The BRISA database now includes over 3,800 HTA reports. Moreover, RedETSA collaborates with various HTA institutions and initiatives, including HTAi, the International Network of Agencies for HTA (INAHTA), the Spanish Network of Agencies for Assessing National Health System Technologies and Performance (RedETS), RedCritieria (a network initiative of the Inter-American Development Bank), HTAsiaLink, and EuroScan.

**Conclusions:**

Significant progress in HTA has been made in the Americas, with RedETSA playing a key role in these achievements. Challenges remain, including establishing explicit links between HTA and decision-making, consolidating institutional frameworks, increasing local data availability, continuing capacity building efforts, and standardizing HTA reports. Continued cooperation between countries and institutions through network collaboration is essential to address these needs.

